# Life satisfaction in UK emerging adults during the COVID-19 pandemic

**DOI:** 10.1007/s12144-023-04580-7

**Published:** 2023-04-11

**Authors:** Christy Lok Yan Li, Leslie Morrison Gutman

**Affiliations:** Department of Clinical, Educational and Health Psychology, University College London, London, WC1E 7HB UK

**Keywords:** Subjective wellbeing, Gender, Mental Health, Young adults, Loneliness, Longitudinal, Emerging adults, Pandemic, COVID-19, Life satisfaction

## Abstract

Current research indicates that young adults are at a higher risk of deteriorating wellbeing during the COVID-19 pandemic compared to older adults. Drawing upon the Understanding Society COVID-19 survey, this study examined the trajectory of life satisfaction in UK emerging adults from May 2020 to September 2021 with social, health, financial, and demographic factors as covariates. The analytic sample included 880 participants (612 females, 268 males) between the ages of 18–29. Growth curve modelling was used to estimate the trajectory of life satisfaction and examine whether the covariates account for variation in the mean level and/or slopes. The trajectory of life satisfaction declined slightly between May 2020 and January 2021 and then increased to September 2021, aligning with the tightening and easing of UK COVID-19 policies. Greater perceived current financial difficulties, pre-existing mental health and physical health conditions, and higher self-reported loneliness were associated with lower life satisfaction. Being female and living with a romantic partner, more face-to-face social interactions, and higher household income were associated with more life satisfaction. Gender interacted with pre-existing mental health conditions. Women with no pre-existing mental health conditions reported the highest level of life satisfaction, while women with pre-existing mental health conditions reported the lowest level, compared to men who reported a similar level of life satisfaction regardless of their mental health. The findings from the present study contribute toward the current understanding of changes in life satisfaction throughout the pandemic among emerging adults. Implications for intervention are discussed.

## Introduction

The ongoing COVID-19 pandemic led to unprecedented disruptions in normal living worldwide. With the long-term social isolation and socio-economic uncertainty resulting from government imposed policies to slow the spread of the virus, significant consequences on subjective wellbeing across populations have been reported (Cheng et al., 2020; Von Soest et al., 2020). The psychological impacts of the pandemic are suggested to be significantly more severe in emerging adults (18 to 29-year-olds) compared to older adults despite the lower likelihood of life-threatening complications, highlighting emerging adults as a potential target population for intervention purposes (Felsenstein & Hedrich, 2020; Sasson, 2021). In particular, life satisfaction in emerging adults has been shown to have declined significantly as the pandemic progressed (Duong, 2021; Preetz et al., 2021). However, scant research has investigated changes in the subjective wellbeing of emerging adults during COVID-19. Existing studies have mostly focused on the initial months of the pandemic or on small samples only (Genç & Arslan, 2021; Preetz et al., 2021; Pretorius & Padmanabhanunni, 2021). Using a UK nationally representative longitudinal data set, the current study addresses this research gap through examining the trajectory of life satisfaction and its associated covariates in emerging adults.

## Emerging Adulthood

Seminally proposed by Arnett et al. (2014), the ages between 18 and 29 are a key developmental period characterised by the exploration of self-identity, career opportunities, interpersonal relationships, and newfound autonomy – known as emerging adulthood. This critical period has been associated with poorer subjective wellbeing and mental health, with anxiety and depressive disorders being especially prevalent (Arnett et al., 2014). This is explained by a focus on entering the workforce and achieving stable independence after adolescence amidst worrying about an uncertain future, which is often accompanied by lower family and social support (Pettit et al., 2011). During the pandemic, opportunities for new relationships were limited by social distancing and developing autonomy was placed on hold as many emerging adults had to return to family homes due to unemployment or online learning, creating a period of great uncertainty and instability. As such, emerging adults may have been particularly vulnerable to psychological maladjustment during the pandemic (Halliburton et al., 2021). For instance, research examining UK emerging adults showed that mental health problems and loneliness increased during lockdowns and eased during periods of fewer restrictions (Hu & Gutman, 2021; Stroud & Gutman, 2021; Thorpe & Gutman, 2022). Specifically, while people have experienced increased anxiety and depressive symptoms due to the pandemic on a population level, young adults appeared to be more sensitive to psychological distress due to their intolerance to uncertainty (Glowacz & Schmits, 2020; Hawes et al., 2021).

## Life satisfaction

Subjective wellbeing refers to how people evaluate and experience different aspects of their own lives (Diener, 1984). Life satisfaction is the most stable component of subjective wellbeing, reflecting one’s overall satisfaction with their life (Diener, 1984). Life satisfaction is considered to be a distinct construct from mental health, as the attitude towards one’s life is measured independent of affective states. Low life satisfaction has been associated with poor outcomes in areas of mental health, physical health, and school attainment (Huebner et al., 2006). For instance, long-term life dissatisfaction is significantly correlated with increased risk of suicide and chronic diseases, positing the maintenance and improvement of life satisfaction as a public health concern (Koivumaa-Honkanen et al., 2001; Rosella et al., 2019). Despite its relevance in fostering psychological strength and protecting against adverse outcomes in many domains (Huebner et al., 2006), there has been a lesser focus on examining the influence of COVID-19 on life satisfaction in comparison to mental health.

Available studies suggest significant changes in life satisfaction associated with the current pandemic. Fancourt et al. (2020) examined life satisfaction in UK emerging adults between March and May 2020, and reported that while life satisfaction increased slightly after lockdown overall, this improvement was less prominent in emerging adults. Additionally, people diagnosed with mental health conditions, in low income households, living alone, and living with children reported comparatively lower life satisfaction. Another study by Preetz et al. (2021) found decreased life satisfaction levels from 2017 to June 2020 in a cohort of German emerging adults. Less peer contact, financial difficulties, and returning to family homes were risk factors for life dissatisfaction in the pandemic, while social integration, having a romantic partner, and self-efficacy were protective factors. However, there appear to be no further studies examining life satisfaction trajectories in emerging adults during the pandemic, especially in the UK.

## Factors associated with life satisfaction in previous studies

### Mental health

Many factors have been correlated with life satisfaction in older adults; however, their relevance in emerging adults during the COVID-19 pandemic has not been fully examined. Research, for example, has consistently established a robust, negative association between life satisfaction and mental health problems (Gigantesco et al., 2019). This relationship is suggested to be reciprocal, where life dissatisfaction significantly influences the development of anxiety disorders, major depressive disorder, substance use disorders, and vice versa (Fergusson et al., 2015). Lopes and Nihei (2021) examined depression, anxiety, and stress symptoms in Brazilian undergraduates in September to October 2020 and found that most participants presented symptoms for internalizing disorders during COVID-19. These symptoms were negatively correlated with life satisfaction and positively correlated with maladaptive coping mechanisms such as substance use. Being a female emerging adult was also identified to be a strong predictor of depression, anxiety, and stress in the study, highlighting a potential at-risk population.

Relatedly, loneliness has been highlighted as a significant non-clinical mental health consequence of the pandemic resulting from social isolation, which led to decreased social contact and support (Hu & Gutman, 2021). Research suggests it is both directly and indirectly associated with life satisfaction, as loneliness was associated with hopelessness and depression in a sample of South African young adults, which in turn related to lower life satisfaction (Padmanabhanunni & Pretorius, 2021). In Turkish older adults, higher levels of loneliness were reported in late 2020 compared to pre-pandemic levels due to prolonged social isolation, which was directly associated with decreases in life satisfaction, although no studies have examined this association for emerging adults (Onal et al., 2022).

### Physical health

Another factor consistently associated with life satisfaction is physical health. Ngamaba et al. (2017) conducted a meta-analysis of 29 studies examining the association between health status and life satisfaction, reporting a significant positive relationship across studies. This has important implications for the pandemic, as having pre-existing health conditions could worsen COVID-19 related anxiety since chronically ill individuals are at a higher risk of severe infections and death (Treskova-Schwarzbach et al., 2021). As such, life satisfaction could be affected due to the decreased quality of life and mental health from increased difficulty accessing healthcare services (Buneviciene et al., 2022; Moynihan et al., 2021). Poor physical health may also be associated with lower life satisfaction for emerging adults during the pandemic. Among Norwegian young adults, for example, poor health was indirectly associated with lowered life satisfaction through worsened work-life balance and job stability worries throughout 2020 to 2021, highlighting the importance of interventions to address and maintain physical health for psychological wellbeing (Bakkeli, 2021).

### Employment and education status

Relatedly, employment has also been associated with life satisfaction. Studies have shown that, regardless of finances and job quality, unemployment is consistently linked to life dissatisfaction, indicating that employment contributes towards life satisfaction by fulfilling productivity and actualization needs (Aysan & Aysan, 2017; Grün et al., 2010; Streimikiene & Grundey, 2009). With COVID-19 labour market instabilities and a global economic recession, recent graduates and emerging adults have been the hardest hit by unemployment (Office for National Statistics, 2021). This points to emerging adults as an at-risk group in need of support, as adults who were unemployed during the early pandemic experienced lower life satisfaction and worse mental health (Zhang et al., 2020). The shift to working from home has had adverse effects due to the loss of a major source of socialization and greater perceived job instability, family distractions, and pandemic-related grief, affecting job performance and in turn inducing psychological distress (Kumar et al., 2021; Nemteanu et al., 2021). This has also been reflected in British undergraduates who had a shift to online learning, reporting dissatisfaction with their work-life balance, wellbeing, and learning experience throughout March 2020 to January 2021 (Maqableh & Alia, 2021). Therefore, employment and education status along with financial security could be important determinants of life satisfaction in emerging adults who should otherwise be furthering education or transitioning into the labour force.

### Demographic factors

Lastly, demographic factors such as gender and ethnicity are suggested to be implicated in differences in life satisfaction, though there are large variations in research. Women on average report higher life satisfaction than men cross-culturally regardless of income, education, and employment levels despite less favourable gender rights globally (Joshanloo & Jovanović, 2020). In a study examining Dutch adolescents after the first lockdown in 2020, while men scored significantly better in mental health than women, they experienced significant decreases in life satisfaction whereas women’s life satisfaction remained unchanged (van der Laan et al., 2021). There are major discrepancies in this finding, with conflicting studies showing either no differences or better life satisfaction in males (Della Giusta et al., 2011). This is suggested to be explained by gender differences in family responsibilities, financial situation, and social engagement, which experienced severe disruptions in the pandemic, thus there might be significant gender differences in life satisfaction as well (Etheridge & Spantig, 2020). Research on life satisfaction differences in ethnic groups has been less prominent, but ethnic minorities are more likely to report lower life satisfaction than Caucasians, especially among immigrants (Safi, 2010). This is explained by perceived discrimination in the host country, which was reflected in anti-Asian sentiments in Western countries during the pandemic (Hahm et al., 2021). Therefore, demographic factors might play a role in life satisfaction experiences during COVID-19. Research on life satisfaction differences in ethnic groups, however, has not been examined. Further understanding is needed to support interventions such as improving access to wellbeing services among ethnic minorities in the UK.

#### Current study

Drawing upon the Understanding Society COVID-19 survey, growth curve modelling (GCM) was used to examine the life satisfaction trajectory among emerging adults from May 2020 to September 2021. Given that young adults are suggested to be disproportionately vulnerable to instabilities and uncertainties in life (Arnett, 2000; Arnett et al., 2014), it is predicted that emerging adults will experience a decrease in life satisfaction overall, with declines aligning with periods of stricter COVID-19 restrictions and improvements with the easing of policies. In line with previous studies (Bakkeli, 2021; Preetz et al., 2021; Lopes & Nihei, 2021; van der Laan et al., 2021), it is further expected that emerging adults who are male and have lower income, more financial insecurity, and pre-existing mental and physical health problems will report lower life satisfaction, while those who are employed and/or in school, have more social contact and live with a romantic partner will report higher life satisfaction. Exploratory interactions between predictors were also tested, with a focus on interactions with gender, although no firm hypotheses are offered as no studies to date have examined these associations during the pandemic for emerging adults.

## Method

### Participants

Participants were from the Understanding Society COVID-19 Survey – a variation of the Understanding Society UK Household Longitudinal Study (UKHLS), a nationally representative longitudinal data set of over 40,000 UK households. Participants were at least 16 years old. Ethical approval was granted by the University of Essex Ethics Committee.

Data from the Understanding Society COVID-19 survey were collected monthly from April to June 2020, bi-monthly from September 2020 to March 2021, and a final wave in September 2021. The first four questionnaires were distributed to participants who have completed at least one questionnaire from the final two waves of the UKHLS, but only those who completed at least one survey from the initial waves were invited from wave 5 (September 2020) onwards. From wave 6 onwards, only those who completed more than one survey since April 2020 were invited again. The Understanding Society COVID-19 Study data are available through the UK Data Service (https://ukdataservice.ac.uk/). The data can be downloaded, ordered or analysed online by registering and accepting their End User Licence.

Data were collected through web surveys and distributed either through emails or SMS. The questionnaire took 20 min to complete, and participants were offered £2 for each survey completed with a £10 incentive for completing the final wave.

Based on the growth curve modelling requirements, the analytic sample included 18-29-year-olds who provided responses for the covariate measures at baseline (April) and reported their life satisfaction at least twice. Of the 880 participants who met these requirements, 30.5% were male, 69.5% female; 84.1% were White, 1.7% Black, 3.6% Mixed, 10.5% Asian, and 0.1% Other.

ANOVAs were conducted to test whether differences between those included (*n* = 880) and those excluded (*n* = 1366) in the analytic sample were significant for the main outcome and covariates. Those who were excluded were more likely to be employed (*F* (1,2069) = 26.47, *p* < .001), attend school (*F* (1,2243) = 12.41, *p* < .001), and live with a romantic partner (*F* (1,2244) = 48.68, *p* < .001) as well as to be male (*F* (1,2240) = 8.28, *p* = .004) and Black (*F* (1,224) = 5.61, *p* = .018) or Asian (*F* (1,2244) = 22.10, *p* < .001) compared to those in the included sample.

## Measures

Table 1 presents the wave, range, mean, and standard deviation of each measure included in the study except for ethnicity and gender. Life satisfaction was measured in 7 out of 9 waves (excluding April and June 2020). Covariate measures were either taken at baseline (January/February 2020) from the main UKHLS or April 2020.

**Table 1:** Descriptive characteristics of measures

Measure	Wave measured	Min.	Max.	Mean	SD
Age	1	18	29	24.26	3.40
Employment	Baseline	0	1	0.80	0.40
Education	Baseline	0	1	0.12	0.32
Physical health condition	Baseline	0	1	0.29	0.02
Mental health condition	Baseline	0	1	0.06	0.24
Face to face social contact	Baseline	1	7	5.19	1.55
Household income	Baseline	1	15	7.14	4.14
Living with partner	1	0	1	0.39	0.49
Loneliness	1	1	3	1.79	0.71
Subjective financial situation	1	1	5	1.95	0.91
LS Wave 2	2	1	7	4.84	1.38
LS Wave 4	4	1	7	5.11	1.36
LS Wave 5	5	1	7	5.04	1.33
LS Wave 6	6	1	7	5.06	1.32
LS Wave 7	7	1	7	4.78	1.46
LS Wave 8	8	1	7	4.98	1.37
LS Wave 9	9	1	7	5.21	1.27

LS = Life satisfaction; SD = Standard deviation

***Ethnicity*** was coded into five categories: White, Mixed, Black, Asian, and Other.

***Gender*** was a dichotomous variable coded as ‘0’ for females and ‘1’ for males.

***Life satisfaction*** was measured from Wave 2 (May 2020) to Wave 9 (September 2021) except for Wave 4 with the single-item question “How satisfied are you currently with your life overall?” (1 = Completely dissatisfied; 7 = Completely satisfied). The validity of using a single question measure of life satisfaction is established in wellbeing research in the UK and US (Fonberg & Smith, 2019).

***Employment status*** was based on the question “Were you in paid work or self-employment at any time in January or February 2020?” (0 = No, 1 = Yes).

***Education status*** was based on a question asking whether the participant was in school instead of work in January or February 2020 (0 = No, 1 = Yes).

***Physical health condition*** was a count of 20 items asking whether participants had any long-term physical health conditions including asthma, heart disease, cancer, epilepsy, or another condition (0 = No, 1 = Yes).

***Mental health condition*** was a dichotomous variable asking whether participants had a long-standing nervous, emotional, or psychiatric condition (0 = No, 1 = Yes).

***Self-reported loneliness*** was based on the question “In the last 4 weeks, how often did you feel lonely?” rated on a 3-point scale (1= “Hardly ever or never”, 2=“Some of the time”, 3=“Often”).

***Current subjective financial situation*** was measured by the question “How well would you say you yourself are managing financially these days?” on a 5-point scale (1 = Living comfortably, 2 = Doing alright, 3 = Just about getting by, 4 = Finding it quite difficult, 5 = Finding it very difficult).

***Household income*** per year was a continuous variable coded with equal intervals of £5000 with a range of 1 to 15 (e.g., 1=£0-£5000, 15=£70,001 and greater).

***Living with a romantic partner*** was a dichotomous variable based on the question “Are you living with a partner?” (0 = No, 1 = Yes).

***Face to face social contact*** was a continuous variable measured by the question “In the last 4 weeks, how often have you met in person with friends and family who do not live with you?” with a range of 1 to 7 (e.g., 1 = Never, 7 = Daily).

### Data analysis

Using SPSS 28, growth curve modelling (GCM) was utilized to examine the trajectory of life satisfaction. GCM does not require equal time points between data and accounts for missing data using the maximum likelihood estimation, which is often a problem in longitudinal research. A time variable was coded according to the number of months passed since Wave 1 to assess changing life satisfaction over the pandemic. A level 1 unconditional model was used to examine changes in life satisfaction across time using both linear and quadratic functions. Then, a level 2 model incorporated covariates to examine their association with life satisfaction at the intercept (Wave 2) and the linear/quadratic slopes. Exploratory interactions were further tested, with a particular focus on interactions with gender. Non-significant interactions are not reported.

## Results

**Fig. 1:**
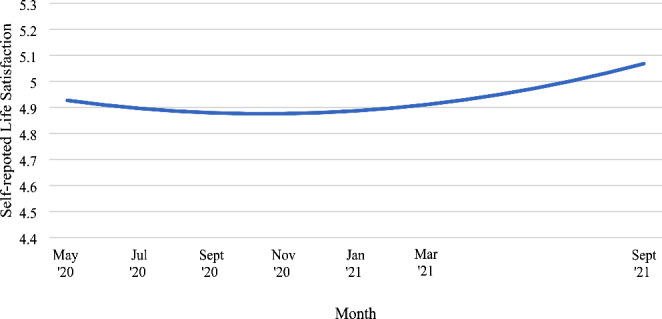
*Trajectory of mean self-reported life satisfaction ratings among UK young adults throughout May 2020 to September 2021*

Table 2 presents the level 1 unconditional model of life satisfaction ratings among UK young adults from May 2020 to September 2021. There was a significant negative linear slope and a significant positive quadratic slope. As shown in Fig. 1, the trajectory of life satisfaction declined slightly between May 2020 and January 2021 and then increased to September 2021.

**Table 2:** Unconditional model examining the changing life satisfaction trajectory

Measure	Coefficient	SE
For intercept		
Intercept	4.95***	0.04
For linear slope		
Intercept	− 0.02**	0.01
For quadratic slope		
Intercept	0.00***	0.00
Residual variance	Variance	
For intercept	0.85***	0.04
For linear slope	0.00*	0.00

***p < .001; **p < .01; *p < .05

Table 3 presents the final model which accounts for variation in the significant covariates. When taking into account the significant covariates, the linear slope was no longer significant but there was a significant positive quadratic slope, indicating non-linear changes in life satisfaction over time. In terms of the covariates, being male, living without a romantic partner and having higher self-reported loneliness, less face-to-face contact with non-household members, more perceived current financial difficulties, lower household income, pre-existing physical health conditions, and pre-existing mental health conditions were significantly associated with lower life satisfaction at the intercept. None of these covariates were significant at the linear/quadratic slopes. Education and employment status, perceived future financial difficulties, and care received outside of the household were not significant predictors at the intercept or linear/quadratic slopes. These covariates were removed from the model for the sake of parsimony. All ethnicity categories were not significant except for mixed-ethnicity participants.

**Table 3:** Growth curve model predicting the life satisfaction trajectory

Measure	Coefficient	SE
For intercept		
Intercept	5.83***	1.7
Male	− 0.29***	0.08
Living with a romantic partner	0.32***	0.06
Income	0.02*	0.01
Current financial difficulties	− 0.30***	0.03
Physical health condition	− 0.13***	0.05
Mental health condition	− 0.59*	0.14
Loneliness	− 0.36***	0.04
Face to face contact	0.06**	0.02
Gender x Mental health	0.64*	0.30
For linear slope		
Intercept	− 0.015	0.01
For quadratic slope		
Intercept	0.00***	0.00
Residual variance	Variance	
For intercept	0.56***	0.04
For linear slope	0.00*	0.00

***p < .001; **p p .01; *p < .05

**Fig. 2:**
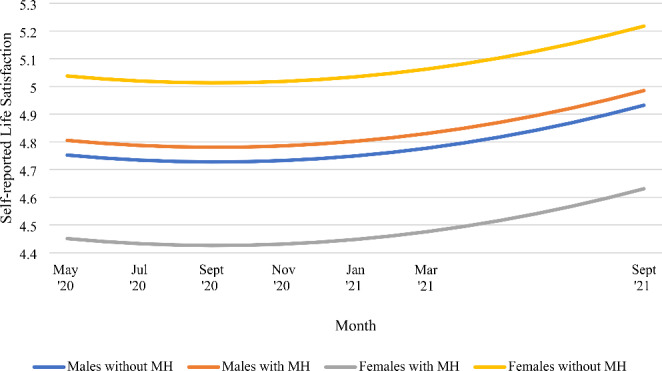
Trajectory of life satisfaction as a function of gender and pre-existing mental health conditions (MH) throughout May 2020 to September 2021

Additionally, gender significantly interacted with mental health (See Fig. 2). Regression equations revealed significant differences in life satisfaction between women with and without mental health conditions, *p* = .034. Women with pre-existing mental health conditions reported the lowest ratings of life satisfaction across all four groups (*M* = 4.48, *SD* = 0.065), while women without pre-existing mental health conditions reported the highest ratings (*M* = 5.07, *SD* = 0.065). There were no significant differences in the life satisfaction ratings between men with (*M* = 4.84, *SD* = 0.065) and without (*M* = 4.78, *SD* = 0.065) mental health conditions.

## Discussion

Drawing upon a nationally representative UK data set, the current study revealed an overall significant decrease in life satisfaction, with the lowest levels in September to November 2020, before an increase beyond baseline levels from March 2021. Being male, living with a romantic partner, loneliness, face-to-face contact with non-household members, household income, perceived financial difficulties, pre-existing mental and physical health conditions were all associated with the mean level of life satisfaction. Additionally, gender interacted with pre-existing mental health conditions. Women with no pre-existing mental health conditions reported the highest level of life satisfaction, while women with pre-existing mental health conditions reported the lowest level, compared to men who reported a similar level of life satisfaction regardless of pre-existing mental health conditions.

### Interpretations and implications of findings

The trajectory of life satisfaction for emerging adults aligned with the tightening and easing of UK COVID-19 policies. In May 2020, participants were only “somewhat satisfied” with their lives on average. This might be due to the ongoing national lockdown and economic downturn from March 2020 which caused disruptions to normal living, likely to be especially severe among emerging adults. This corroborates with previous research reporting lower occupational activity, less living space, and less social contact among emerging adults during the pandemic, which could lead to lower life satisfaction due to intolerance to uncertainty (Glowacz & Schmits, 2020). Although most restrictions were lifted in July 2020, the trajectory continues to decline with the trough around September to November 2020 when the second wave of COVID-19 began in the UK. This aligns with the tightening of restrictions over these two months, including the ‘rule of six’ to curb large social gatherings on September 14th, the return to work from home on September 22nd, and another nationwide lockdown on November 5th (Institute for Government Analysis, 2022). This decline might stem from pandemic fatigue and hopelessness as the UK underwent another lockdown after normal living in the summer months, resulting in more distress compared to the first lockdown (Moradian et al., 2021). Despite imposing a third lockdown in January 2021, life satisfaction steadily increased after November 2020. This could reflect adaptation to the “new normal” as young adults navigate through work from home settings and socialising in the pandemic, regaining consistency in their lives (Corpuz, 2021). Moreover, vaccinations were offered to all adults by July 2021. Research shows that psychological stress decreased after vaccinations, likely due to fewer COVID-19 concerns and increased socializing opportunities for emerging adults (Perez-Arce et al., 2021). Therefore, life satisfaction of emerging adults seems to be aligned with the government policies imposed in the UK.

Furthermore, the social stigma surrounding protective measures (e.g. face masks) in Western countries might also contribute to the trends in life satisfaction ratings among UK emerging adults in the initial months of the pandemic. While compulsory mask-wearing policies were quickly enforced in Asian countries such as China to control the spread of the virus, medical experts from the UK actively advised against the use of face masks among the healthy general public due to a lack of consistent evidence in the airborne transmission of COVID-19 at the time (Feng et al., 2020). This led anti-mask sentiments to be widespread among the UK population, and mask-wearing signalled an embodied stigma often leading users to face discrimination, especially ethnic minorities (Xiao et al., 2020). As a result, only 28% of adults reported having used a face covering in May 2020 in a UK nationwide survey (Office for National Statistics, 2021). Lower rates of face mask use have been associated with negative psychological impacts in countries that discouraged face mask use. For instance, compared to China where face masks in public were mandatory, Polish citizens reported significantly higher levels of depression, anxiety, and stress (Wang et al., 2020). This was explained by Polish participants reporting significantly more COVID-19 symptoms and hospitalization rates, possibly leading to increased COVID-19-related distress and deteriorating wellbeing compared to Chinese participants. As such, the similar lack of consistent public health information and disuse of effective precautionary measures in the UK during the early months of the pandemic may have contributed to the declining life satisfaction trajectory in the current study. However, despite the subsequent mask mandate in July 2020, life satisfaction ratings continued to decline until November 2020, indicating that the destigmatization of mask-wearing is not a major deciding factor in life satisfaction among UK emerging adults. Nonetheless, reliable and consistent health education is an important factor in preventing the spread of the virus and misinformation, thus should be provided by government sources as the pandemic evolves to protect against negative psychological impacts.

At-risk subgroups for lower life satisfaction in the pandemic were identified. Emerging adults with pre-existing mental health conditions reported lower life satisfaction compared to individuals without diagnoses, reaffirming the association between mental health and life satisfaction (Lombardo et al., 2018; Lopes & Nihei, 2021). This could stem from stress from higher uncertainty, and greater relapse risk due to overwhelmed treatment services, making them more vulnerable to COVID-19 related stressors, hence lowering life satisfaction (Chatterjee et al., 2020). Moreover, an association was found between loneliness and life satisfaction (Onal et al., 2022). Therefore, interventions should address the reduced levels of social interaction and place equal importance on non-clinical mental distress among emerging adults, as this is a critical period for interpersonal and social development (Arnett, 2000).

Face-to-face contact with non-household members and living with a romantic partner were associated with better life satisfaction in the pandemic, corroborating with past research (Amati et al., 2018; Næss et al., 2015). As lockdown restricts in-person interactions to household members, a romantic partner could provide a source of emotional support in times of stress, enhancing life satisfaction (Tsang et al., 2022). Face-to-face socializing might have transferred into internet-mediated socializing to maintain life satisfaction, though online socializing has been shown to be less beneficial to wellbeing than in-person interactions (Foulkes & Blakemore, 2021; Simone et al., 2019). Therefore, optimizing social support policies and directing resources towards social befriending services might improve life satisfaction.

Pre-existing physical health conditions were also significantly associated with lower life satisfaction, consistent with existing research (Ngamaba et al., 2017). This is understood by heightened COVID-19 related anxiety since chronically ill individuals are at higher risk of severe infections compared to healthy young adults if they contract the virus (Treskova-Schwarzbach et al., 2021). As such, decreased quality of life and lower mental health related to difficulty accessing healthcare services could lower life satisfaction (Buneviciene et al., 2022; Moynihan et al., 2021). Resources should be directed towards supporting the psychological wellbeing of emerging adults with physical health conditions, whose lives are already more disrupted than their healthy peers pre-pandemic. However, the varying impacts of different illnesses and the association between COVID-19 symptoms experienced and life satisfaction remain to be explored in future research.

Similarly, associations between financial security and lower life satisfaction in emerging adults were found, in terms of both household income and perceived current financial situation. This might be explained by the uncertainty and pandemic threat in society, causing increased importance on material goals (Zheng et al., 2021). This effect is magnified in emerging adults, as they experience higher unemployment rates, thus the role of financial security to satisfy needs is emphasised. Therefore, increasing unemployment benefits and stimulus checks might contribute towards enhanced life satisfaction in the pandemic.

Being male was also associated with lower life satisfaction. This can be attributed to gender differences in determinants of life satisfaction, as men are found to value socio-political, employment, and education related variables more in life satisfaction while women value interpersonal relationships and marital status more (Joshanloo, 2018). This would explain gender differences during COVID-19, as there was socio-political instability, and education and employment were severely disrupted among emerging adults. Meanwhile, women tend to have larger social networks and more social support (Antonucci & Akiyama, 1987), and socializing can be satisfied through online means, maintaining their life satisfaction. The gender and mental health interaction was significant, showing that women with pre-existing mental health problems were most at risk of life dissatisfaction. This might be explained by the affect intensity rationale, which suggests women’s life satisfaction is more affected by both positive and negative emotions than men’s (Becchetti & Conzo, 2022; Diener et al., 1985). This rationale could explain why women without mental health conditions had higher life satisfaction than men with or without mental health conditions, but women with mental health conditions reported lower life satisfaction than men in both conditions. Overall, results emphasise the need to increase resources towards overwhelmed mental health services in the UK. Further research examining whether there are gender differences in the association between specific pre-existing mental health conditions and life satisfaction during COVID-19 is warranted to better inform target populations for interventions.

#### Limitations

Although the Understanding Society COVID-19 Survey is nationally representative, the final analytic sample was limited. For instance, the analytic sample was overrepresented by white participants and females, lacking representation of ethnic minorities and males. This applies to covariates which significantly differed between the included and excluded sample, which may have been significant with greater representation. Moreover, not all participants responded to all waves of data collection, resulting in missing data. This restricted direct comparisons of life satisfaction changes. Nonetheless, the use of growth curve modelling and maximum likelihood estimations allows trajectories to be analyzed with only two data points, meaning that missing data can be accounted for.

Secondly, the results are correlational – causal relationships between covariates and changing life satisfaction cannot be inferred. Nonetheless, correlational results still provide insight into factors influencing life satisfaction in the pandemic since factors cannot be manipulated on a large scale. Furthermore, the Understanding Society COVID-19 Survey first collected life satisfaction data in May 2020; therefore, the current study could not examine how the trajectory of life satisfaction changed since before the pandemic or the first UK lockdown in March 2020, limiting the conclusions of the current study. Moreover, covariate measures were only taken at baseline or April 2020 to predict life satisfaction throughout the next 17 months, but the changing covariate trajectories throughout the pandemic could have influenced life satisfaction as well. For instance, employed participants at baseline could become unemployed later due to the economic recession, which might lower life satisfaction. Therefore, future studies should track covariate trajectories and examine whether they interact to influence the life satisfaction trajectory.

Lastly, life satisfaction and loneliness were investigated by a single-item measure, lacking the complexity to achieve a complete understanding. For instance, the distinction between social and emotional loneliness cannot be reflected in the single-item measure (Weiss, 1973). Subjective ratings were also used, meaning that what one considers as “often” lonely could be judged as “some of the time” to another, hence measures could have been inaccurately reported. Moreover, the single-item measure of life satisfaction is considered to be more prone to measurement errors and to have lower stability in longitudinal research compared to the multiple-item Satisfaction With Life Scale (SWLS), though one study reported similar test-retest reliability between the two measures when compared and examined over 10 months among a large Serbian sample (Jovanović & Lazic, 2020). The single-item measure of life satisfaction has also been validated in research (Fonberg & Smith, 2019) and the single-item measure of loneliness was recommended by the Office for National Statistics (2018) in the UK, supporting their use in the current study. Future research could consider adding multiple-item scales, such as the SWLS, in combination with the single-item question to fully capture the nuances within these measures. In addition, while mental health diagnosis was objectively examined through a single question, this does not capture data from individuals who have not been diagnosed but would qualify otherwise, or those who experience symptoms but not severe enough for clinical diagnosis. Other covariate measures that lack complexity include employment (type of job could impact life satisfaction), living with a partner (long-term confinement with unsupportive partners could worsen life satisfaction instead), and face-to-face contact (the quality of social support could influence life satisfaction). Therefore, future replications of the current study addressing the limitations listed are needed.

## Conclusion

The current study highlights the impact of COVID-19 on the life satisfaction of emerging adults. The trajectory modelled aligned with the imposing and lifting of restrictions, confirming that subjective wellbeing is affected by pandemic conditions and public health interventions are direly needed. The findings highlight that females with pre-existing mental health conditions are an especially vulnerable group. Targeted interventions for emerging adults who are identified as at-risk are needed in pandemic conditions, especially during strict restrictions. Based on the findings, this might include promoting online social events in schools or companies, and directing resources towards healthcare and financial aid to mitigate adverse outcomes in life satisfaction. Overall, the current study charted the life satisfaction trajectory of UK young adults using longitudinal data and identified significant predictors, clarifying and contributing to the developing field of COVID-19 research.

## Data Availability

UK Data Service.
